# Selection of chromosome segment substitution lines with reduced grain chalkiness without yield penalty in rice

**DOI:** 10.1270/jsbbs.24044

**Published:** 2025-03-22

**Authors:** Hirofumi Fukuda, Akari Fukuda, Yasunori Nonoue, Di Guan, Noriko Kanno, Shoji Taniguchi, Ryoji Imase, Kei Matsushita, Sota Kitasaki, Yuzo Komaki, Minoru Takemure, Toshihiro Sakamoto, Shuichi Fukuoka, Jun-ichi Yonemaru, Daisuke Ogawa

**Affiliations:** 1 Institute of Crop Science, National Agricultural and Food Research Organization (NARO), Tsukuba, Ibaraki 305-8518, Japan; 2 Research Center for Agricultural Information Technology, National Agricultural and Food Research Organization (NARO), Tsukuba, Ibaraki 305-0856, Japan; 3 Toyama Prefectural Agricultural, Forestry & Fisheries Research Center, 1124-1 Yoshioka, Toyama, Toyama 939-8153, Japan; 4 Kagoshima Prefectural Institute for Agricultural Development, 2200 Ono, Kinpo, Minamisatsuma, Kagoshima 899-3401, Japan; 5 Institute for Agro-Environmental Sciences, National Agricultural and Food Research Organization (NARO), Tsukuba, Ibaraki 305-8604, Japan

**Keywords:** advanced mapping population, agronomic trait, CSSL, *Oryza sativa*, perfect grain

## Abstract

Grain chalkiness decreases the appearance quality (APQ) of rice (*Oryza sativa* L.) grains and reduces consumer satisfaction. Improving APQ is a crucial issue for both marketing and breeding. Here, we screened chromosome segment substitution lines (CSSLs) with higher APQ to find promising genetic resources. These CSSLs harbor chromosome segments derived from multiple donors in the genetic background of ‘Koshihikari’, a leading *japonica* rice cultivar in Japan. Three CSSLs had an increased percentage of perfect grains without panicle weight loss under field conditions across 3 years in Tsukuba city, Ibaraki prefecture, Japan. The positions of reduced chalkiness in grains differed among CSSLs, suggesting the different contribution of the harbored chromosome segments to APQ improvement. There were no significant differences in days to heading, culm length, panicle length, or panicle number in all three CSSLs, but 1000-grain weight was reduced in one. These results identify two promising genetic resources for further improvement of APQ in current *japonica* cultivars with reduced chalkiness but unaltered heading date and yield traits.

## Introduction

The appearance quality (APQ) of rice grains is a highly complex trait which can be strongly affected by heading date, and storage compounds in grains (Chen *et al.* 2013, Gong *et al.* 2020, Ishimaru *et al.* 2020, Yamakawa *et al.* 2008). APQ is assessed by four major properties, namely grain shape, chalkiness, transparency, and grain color. Their degree of importance to APQ depends on consumer preferences. Among them, chalkiness commonly decreases APQ, especially in temperate countries. Chalkiness is indicated by opaque parts in the milled rice grain caused by the abnormal deposition of starch and storage proteins in the endosperm (Wang *et al.* 2008, Yoshioka *et al.* 2007, reviewed by Zhao *et al.* 2022). It is categorized by the position of the chalkiness as white basal, white belly, white back, white core, or white milky. Several quantitative trait loci (QTLs) control chalkiness (Kobayashi *et al.* 2013, Takehara *et al.* 2018, Yamakawa *et al.* 2008) with changes of days to heading (DTH), grain length-to-width ratio, or 1000-grain weight (TGW) (i.e. grain yield). Further investigation of QTLs that improve APQ without yield penalty is still required.

Most QTL mapping studies in rice generally rely on primary mapping populations such as recombinant inbred lines (RILs) but can fail to identify QTLs with subtle effects, owing to diminished sensitivity of detection due to the use of early generations (Kobayashi *et al.* 2013, Yamamoto *et al.* 2009). Conversely, advanced mapping populations can break through this limitation since their genetic backgrounds are more genetically fixed, resulting in higher sensitivity of detection (Nagata *et al.* 2015, Tanksley and Nelson 1996, Yamamoto *et al.* 2009). For example, chromosome segment substitution lines (CSSLs) were used to detect novel QTLs for preharvest sprouting resistance, DTH, culm length (CL), yield traits, toxic metal concentrations, and APQ components have been uncovered (Abe *et al.* 2013, Bian *et al.* 2010, Hao *et al.* 2009, Hori *et al.* 2010, Ishikawa *et al.* 2005, Nagata *et al.* 2023, Takai *et al.* 2014, Yamakawa *et al.* 2008).

Significant progress has been made in developing CSSLs with the genetic background of ‘Koshihikari’, a leading *japonica* cultivar in Japan, which carry chromosome segments from the *indica* cultivar ‘Habataki’ (Murata *et al.* 2014). However, CSSLs carrying chromosome segments from other *indica* (*indica* and *aus*) in addition to *tropical japonica* donors were not well screened on the purpose of increasing APQ in the ‘Koshihikari’ genetic background. Thus, evaluating the chalkiness or percentage of perfect grains (PPG) among such CSSLs may highlight the promising genetic resources with a high APQ under the present cultivation system for ‘Koshihikari’.

To evaluate chalkiness, we selected CSSLs containing chromosome segments from *indica*, *aus*, and *tropical japonica* cultivars in the genetic ‘Koshihikari’ background (Ishikawa *et al.* 2005, Nagata *et al.* 2015, 2023). Many agronomic traits of these materials vary widely from those of ‘Koshihikari’, but chalkiness and PPG have not yet been examined. We screened CSSLs with a similar DTH to ‘Koshihikari’ to minimize the effects of environmental factors on grain traits and found three CSSLs with reduced chalkiness and higher PPG without loss of panicle weight (PW) under field conditions across 3 years.

## Materials and Methods

### Plant materials and growth conditions

The 342 CSSLs, developed in previous studies (Ishikawa *et al.* 2005, Nagata *et al.* 2015, 2023), possess chromosome segments of *indica* (*indica*: ‘Bei Khe’, ‘Bleiyo’, ‘IR64’, ‘Naba’, ‘Tupa 121-3’), *aus* (‘Kasalath’, ‘Muha’) and *tropical japonica* (‘Basilanon’) cultivars in the ‘Koshihikari’ genetic background. All plants were grown under field conditions in Kannondai, Tsukuba city, Ibaraki prefecture, Japan, in 2019–2021 (36°01ʹ29.4ʺN, 140°06ʹ28.4ʺE, 21 m a.s.l.) and 2023 (36°01ʹ28.7ʺN, 140°06ʹ37.3ʺE, 23 m a.s.l.). The average temperature and relative humidity in our field were measured at 2 m above the ground (Supplemental Fig. 1). More details of the climatic conditions are available from https://www.naro.affrc.go.jp/org/niaes/aws/weatherdata.html (in Japanese). Seeds were soaked in water at 30°C for 2 d, sown in trays filled with soil and incubated at 30°C in the dark for 2 d; seeds were sown on 22 April 2019, 20 April 2020, 19 April 2021, and 26 April 2023. Seedlings were grown in a paddy field for a month and were then transplanted (9 or 11 plants, 18 cm apart × 3 rows 30 cm apart, no replicates) into a nearby paddy field, and grown for 5 months from May to September. Planting density was 18.5 hills/m^2^. Fertilizer was applied as basal dressing at a rate of 56:16.4:56:72:120 kg/ha for N:PO_4_:K:Mg:SiO_2_ in 2019, 2020, and 2021; basal dressing at a rate of 60:60:45:15:60 kg/ha for (NH_4_)_2_SO_4_:P_2_O_5_:K_2_O:Mg in 2023. Other cultivation conditions in different cities of Ibaraki prefecture, Toyama prefecture, and Kagoshima prefecture are described in Supplemental Table 1.

### Assessment of agronomic traits of rice plants

Agronomic traits were measured as previous studies (Ogawa *et al.* 2021a, 2021b). DTH was scored as the number of days from sowing to the appearance of the first panicle in more than half of the plants in each line. CL and panicle length (PL) of the longest culm on each plant were measured with a ruler, and panicle number (PN) was counted, from 10 days to a month after heading. For measurement of aboveground dry weight (ADW), PW, and stem and leaf weight (SLW), shoots of mature plants were dried for over a month and cut 3 cm below the panicle base to separate the parts. Five plants were evaluated per line for CL, PL and PN. Grain weight (GW) was measured after threshing. TGW was measured after hulling in only Kannondai.

To evaluate APQ, we used a 96-dpi scanned image of >400 seeds per individual plant, and 4 plants per line as biological replicates in Kannondai in 2023. Seeds were obtained from panicle bulks, and calculated PPG with a grain discriminator (RGQI90A or RGQI100B, Satake). Perfect grains were defined as non-chalky, with a normal shape (Ebitani *et al.* 2008, Takehara *et al.* 2018). Grain length (GL), width (GW), and thickness (GT) were measured by the latter grain discriminator. Each trait value of ‘Koshihikari’ was calculated as the mean of all ‘Koshihikari’ plants grown at the same time under the same conditions.

## Results

### Characterization of target CSSLs with similar DTH to ‘Koshihikari’

The target CSSLs (148 lines) were defined as having DTH within the range of ‘Koshihikari’ maximum +1 to minimum –1 in 2019, 2020, and 2021. DTH in ‘Koshihikari’ were 101–104 in 2019, 98–101 in 2020, and 104–106 in 2021 (Supplemental Table 2). In 2020 and 2021, we investigated the AGW, SLW, CL, PW, PL, and PN of the target CSSLs (Supplemental Fig. 2, Supplemental Table 2). The CSSLs had biomass and yield trait values ranging from –37% to +16% of ‘Koshihikari’ in AGW, –24% to +22% in SLW, –24% to +16% in CL, –36% to +18% in PW, –13% to +18% in PL, and –34% to +19% in PN. PPG ranged from –98% to +96% of ‘Koshihikari’. This motivated us to search for promising CSSLs having higher APQ without yield penalty.

### Selection of promising CSSLs with higher PPG in 2020 and 2021

**Fig. 1.**
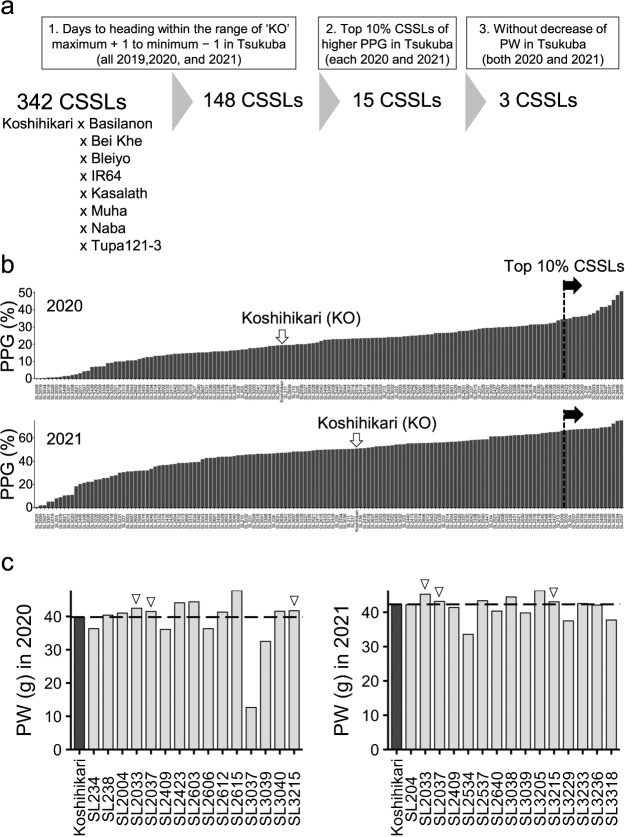
Selection of promising lines with higher percentage of perfect grains without panicle weight loss. (a) Selection of three promising lines with a higher percentage of perfect grains (PPG) without panicle weight loss from 342 chromosome segment substitution lines (CSSLs) with the ‘Koshihikari’ (KO) genetic background under field conditions in 2020 and 2021. (b) PPG was assessed in 148 CSSLs with days to heading within the range of ‘Koshihikari’ ± 1 day in Tsukuba in 2020 and 2021. Dashed line marks the 90^th^ percentile of PPG among CSSLs, which was exceeded by 15 lines in each year. (c) Panicle weights (PW) of the 15 top CSSLs in *b*. ▽ Three promising lines with a higher PPG without PW loss under the field conditions in 2020 and 2021.

To screen lines with higher PPG but without yield penalty from the 148 target CSSLs, we selected the top 10% (15 CSSLs) by PPG in 2020 and 2021 (Fig. 1a, 1b) and compared PW between each CSSL and ‘Koshihikari’ in the same years. We found three promising CSSLs (SL2033, SL2037, and SL3215) with no decreased PW in both 2020 and 2021 (Fig. 1c).

By comparing the locations of the introgressed chromosome segments between the three promising CSSLs (Nagata *et al.* 2015, 2023) and ‘Koshihikari’, we found introgressed chromosome segments between 1 × 10^–6^ and 13.45 Mb on chromosome (Chr.) 10 in SL2033 (carrying ‘IR64’ chromosome segment(s)); between 1 × 10^–6^ and 4.06 Mb on Chr. 11 in SL2037 (carrying ‘IR64’ chromosome segment(s)); and between 1 × 10^–6^ and 19.26 Mb, and between 28.42 and 29.75 Mb on Chr. 5 in the SL3215 (carrying ‘Naba’ chromosome segments) (Table 1).

### Changes in appearance quality of rice grains in promising CSSLs

**Fig. 2.**
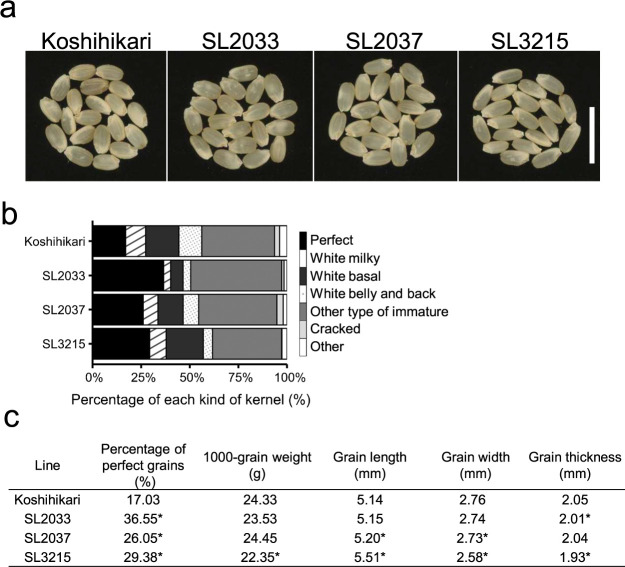
Appearance quality of brown rice in promising CSSLs harvested in 2023. (a) Grains of a rice plant cultivated under experimental filed conditions in 2023. Three promising CSSLs (SL2033, SL2037, SL3215) exhibited higher PPG compared to ‘Koshihikari’ in 2023. Grains of ‘Koshihikari’ exhibit more chalkiness in a wide range and less transparency. Scale bar, 10 mm. (b) Percentage of different kinds of kernel in the three lines and ‘Koshihikari’. (c) Percentage of perfect grains, average of grain volume-related traits (1000-grain weight, grain length, grain width, and grain thickness) in the three lines (SL2033, SL2037, SL3215) and ‘Koshihikari’. Asterisks indicate significant differences between each CSSL and the genetic ‘Koshihikari’ background (n = 4, *P* < 0.05, Dunnett’s Test).

To understand more about the chalkiness phenotypes, we analyzed and compared the ratios of different kinds of kernels among SL2033, SL2037, SL3215, and ‘Koshihikari’. All three CSSLs had lower proportions of white milky, white belly, and white back grains than ‘Koshihikari’, increasing PPG (Fig. 2). SL2033 and SL2037 also had lower proportions of white basal grains (Fig. 2b). These results implicate the different effects on grain chalkiness among the introgressed chromosome segments harbored in each CSSL.

Next, we investigated the differences in TGW, GL, GW, and GT between each promising CSSL and ‘Koshihikari’ in 2023. SL2033 had significantly lower GT (*P* < 0.05); SL2037 had significantly longer GL and lower GW; and SL3215 had significantly lower TGW, longer GL, lower GW, and lower GT (Fig. 2c). The other grain phenotypes not described for each CSSL were not significantly different from those of ‘Koshihikari’. These results indicate that SL2033 produced flatter grains; SL2037 produced slenderer grains; and SL3215 produced lighter, slenderer, and flatter grains.

### Investigation of the growth phenotypes of culms and panicles in promising CSSLs

There were no significant differences in CL, PL, PN, or PW between each CSSL and ‘Koshihikari’ in both 2020 and 2021 (Supplemental Fig. 3).

### Percentage of perfect grains and yield in promising CSSLs under field conditions in different Japanese cities

To investigate the characteristics of SL2033, SL2037, and SL3215 in terms of PPG and yield in other Japanese sites, we cultivated them under field conditions in Tsukubamirai (Ibaraki prefecture), Toyama (Toyama prefecture), and Minamisatsuma cities (Kagoshima prefecture) in 2023 (Supplemental Tables 3, 4). In Tsukubamirai city, all the three CSSLs exhibited a higher PPG and yield (kg/ha) compared to ‘Koshihikari’ as same as in Tsukuba city. In Toyama city, SL2033 and SL2037 exhibited higher PPG, but all the three CSSLs exhibited lower yield compared to ‘Koshihikari’. In Minamisatsuma city, SL2037 and SL3215 exhibited higher PPG, but only SL2037 exhibited higher yield compared to ‘Koshihikari’. These results implicate that performance of the three CSSLs are different depending on the environment conditions despite the qualitatively similar performance in/near Tsukuba city.

## Discussion

We screened genetic resources for reduced grain chalkiness. To minimize the impact of environmental factors, we selected the 148 target CSSLs with comparable DTH to that of ‘Koshihikari’ from 342 CSSLs in which chromosome segments of *indica*, *aus*, or *tropical japonica* rice donors are introgressed into the ‘Koshihikari’ genetic background. From the 148 CSSLs, we isolated SL2033 and SL2037, with reduced grain chalkiness without large effects on yield traits; and SL3215, with reduced grain chalkiness, and yield loss due to reduced grain size, under field conditions in Ibaraki prefecture. Although the performance of the three CSSLs were not qualitatively consistent in different environmental conditions, each of them still exhibited higher PPG than ‘Koshihikari’ in different sites across two or three prefectures.

Here, we will discuss the candidate QTLs related to APQs in finally selected CSSLs in this study. SL3215 has chromosome segments at 1 × 10^–6^–19.26 Mb and 28.42–29.75 Mb on Chr. 5 introgressed from ‘Naba’; SL2033 has a segment at 1 × 10^–6^–13.45 Mb on Chr. 10 from ‘IR64’; and SL2037 has a segment at 1 × 10^–6^–4.06 Mb on Chr. 11 from ‘IR64’ (Nagata *et al.* 2015, 2023). Several QTLs enhancing APQ have been reported on two of these same chromosomes. Ebitani *et al.* (2008) detected ‘Kasalath’ alleles of QTLs on Chrs. 2, 9, 11, and 12. Although we used ‘Kasalath’ as a donor in our CSSLs, CSSLs with introgressed chromosome segments from ‘Kasalath’ were not selected here because of their lower PWs than ‘Koshihikari’ and different DTH. As the introgressed region in SL3215 has a well-known chalkiness-related QTL, *CHALKINESS 5* (LOC_Os05g06480) (Li *et al.* 2014), it is highly likely that *Chalk5* is the causal gene for the improved APQ in SL3215. Two well-known QTLs related to grain weight and size, *GRAIN SIZE AND NUMBER 1* (LOC_Os05g02500) and *SMALL AND ROUND SEED 3* (LOC_Os05g06280) (Guo *et al.* 2018, Ngangkham *et al.* 2018), in the same region are candidates for loci conferring smaller grain size in SL3215. To the best of our knowledge, SL2033 and SL2037 have no known QTLs enhancing APQ in the introgressed chromosome segments.

It may be possible to consider the other candidate genes from the viewpoint of mechanisms affecting chalkiness. For example, preventing sucrose from being degraded and from being used for cellulose biosynthesis in stems enhances APQ of rice grains, because the transport of more sucrose to the endosperm facilitates the synthesis of storage components and results in reduced chalkiness (Shi *et al.* 2024). The ‘Naba’ segment on Chr. 5 in SL3215 harbors *POLYGALACTURONASE-INHIBITING PROTEIN*s *1* to *4* (Os05g0104200 / 4150 / 4600 / 4700) and *RICE STARCH REGULATOR 1* (Os05g0121600). The ‘IR64’ segment on Chr. 10 in SL2033 harbors *CELLULOSE SYNTHASE LIKE H1* and *F7* (Os10g0341401 / 3400). That on Chr. 11 in SL2037 harbors *PECTIN METHYLESTERASE INHIBITOR*s *37* to *39* (Os11g0132100 / 2300 / 2550), *CHALCONE ISOMERASE-LIKE 1* (Os11g0116300) (Lam *et al.* 2022), and *ARABINOFURANOSIDASE 3* (Os11g0131900) (Sumiyoshi *et al.* 2013). These genes, related to cellulose metabolism or starch accumulation, might be potential candidates for reduced chalkiness in our selected CSSLs.

This study revealed several promising genetic resources available to enhance APQ of rice grains under the field conditions in Japanese sites. Further study revealing how the APQ is enhanced in the finally selected CSSLs will be required to maximize the benefit of introgressed chromosome segments / undetected QTLs when performing gene pyramiding in the future.

## Author Contribution Statement

DO conceptualized the research. HF, AF, YN, DG, NK, KM, SK, YK, MT, ST, RI, TS, JY and DO performed the investigations. SF provided the resources. HF and DO performed data curation. JY and DO helped with funding acquisition. HF wrote the manuscript. SF and DO edited the manuscript. All authors have reviewed drafts of the paper and approved the final draft.

## Supplementary Material

Supplemental Figures

Supplemental Table 1

Supplemental Table 2

Supplemental Table 3

Supplemental Table 4

## Figures and Tables

**Table 1.** Positions of chromosome segment substitutions in the selected CSSLs

CSSL	Donor			Chr.	Range of target chromosome per line (Mb)
	Variety name	Origin	Subspecies		
SL2033	IR64	Philippines	*indica*	10	1 × 10^–6^–13.45
SL2037	IR64	Philippines	*indica*	11	1 × 10^–6^–4.06
SL3215	Naba	India	*indica*	5	1 × 10^–6^–19.26, 28.42–29.75

## References

[B1] Abe, T., Y. Nonoue, N. Ono, M. Omoteno, M. Kuramata, S. Fukuoka, T. Yamamoto, M. Yano and S. Ishikawa (2013) Detection of QTLs to reduce cadmium content in rice grains using LAC23/Koshihikari chromosome segment substitution lines. Breed Sci 63: 284–291. 24273423 10.1270/jsbbs.63.284 PMC3770555

[B2] Bian, J.M., L. Jiang, L.L. Liu, X.J. Wei, Y.H. Xiao, L.J. Zhang, Z.G. Zhao, H.Q. Zhai and J.M. Wan (2010) Construction of a new set of rice chromosome segment substitution lines and identification of grain weight and related traits QTLs. Breed Sci 60: 305–313.

[B3] Chen, C., J. Huang, L. Zhu, F. Shah, L. Nie, K. Cui and S. Peng (2013) Varietal difference in the response of rice chalkiness to temperature during ripening phase across different sowing dates. Field Crops Res 151: 85–91.

[B4] Ebitani, T., Y. Yamamoto, M. Yano and M. Funane (2008) Identification of quantitative trait loci for grain appearance using chromosome segment substitution lines in rice. Breed Res 10: 91–99 (in Japanese).

[B5] Gong, R., D. Huang, Y. Chen, H. Li, Z. Wang, D. Zhou, L. Zhao, Y. Pan, Y. Chang, Y. Xiang et al. (2020) Comparative metabolomics analysis reveals the variations of eating quality among three high-quality rice cultivars. Mol Breed 40: 112.

[B6] Guo, T., K. Chen, N.Q. Dong, C.L. Shi, W.W. Ye, J.P. Gao, X.J. Shan and H.X. Lin (2018) *GRAIN SIZE AND NUMBER_1_* negatively regulates the OsMKKK10-OsMKK4-OsMPK6 cascade to coordinate the trade-off between grain number per panicle and grain size in rice. Plant Cell 30: 871–888. 29588389 10.1105/tpc.17.00959 PMC5973843

[B7] Hao, W., M.Z. Zhu, J.P. Gao, S.Y. Sun and H.X. Lin (2009) Identification of quantitative trait loci for rice quality in a population of chromosome segment substitution lines. J Integr Plant Biol 51: 500–512. 19508361 10.1111/j.1744-7909.2009.00822.x

[B8] Hori, K., K. Sugimoto, Y. Nonoue, N. Ono, K. Matsubara, U. Yamanouchi, A. Abe, Y. Takeuchi and M. Yano (2010) Detection of quantitative trait loci controlling pre-harvest sprouting resistance by using backcrossed populations of *japonica* rice cultivars. Theor Appl Genet 120: 1547–1557. 20145904 10.1007/s00122-010-1275-z PMC2859223

[B9] Ishikawa, S., N. Ae and M. Yano (2005) Chromosomal regions with quantitative trait loci controlling cadmium concentration in brown rice (*Oryza sativa*). New Phytol 168: 345–350. 16219074 10.1111/j.1469-8137.2005.01516.x

[B10] Ishimaru, T., M. Miyazaki, T. Shigemitsu, M. Nakata, M. Kuroda, M. Kondo and T. Masumura (2020) Effect of high temperature stress during ripening on the accumulation of key storage compounds among Japanese highly palatable rice cultivars. J Cereal Sci 95: 103018.

[B11] Kobayashi, A., J. Sonoda, K. Sugimoto, M. Kondo, N. Iwasawa, T. Hayashi, K. Tomita, M. Yano and T. Shimizu (2013) Detection and verification of QTLs associated with heat-induced quality decline of rice (*Oryza sativa* L.) using recombinant inbred lines and near-isogenic lines. Breed Sci 63: 339–346. 24273430 10.1270/jsbbs.63.339 PMC3770562

[B28] Lam, P., Y.L. Wang, A.C. Lui, H Liu, H.Y. Takeda-Kimura, M.X. Chen, F.Y. Zhu, J. Zhang, T. Umezawa, Y. Tobimatsu et al. (2022) Deficiency in flavonoid biosynthesis genes *CHS*, *CHI*, and *CHIL* alters rice flavonoid and lignin profiles. Plant Physiol 188: 1993–2011. 34963002 10.1093/plphys/kiab606 PMC8969032

[B12] Li, Y., C. Fan, Y. Xing, P. Yun, L. Luo, B. Yan, B. Peng, W. Xie, G. Wang, X. Li et al. (2014) *Chalk5* encodes a vacuolar H^+^-translocating pyrophosphatase influencing grain chalkiness in rice. Nat Genet 46: 398–404. 24633159 10.1038/ng.2923

[B13] Murata, K., Y. Iyama, T. Yamaguchi, H. Ozaki, Y. Kidani and T. Ebitani (2014) Identification of a novel gene (*Apq1*) from the *indica* rice cultivar ‘Habataki’ that improves the quality of grains produced under high temperature stress. Breed Sci 64: 273–281. 25914581 10.1270/jsbbs.64.273 PMC4267301

[B14] Nagata, K., T. Ando, Y. Nonoue, T. Mizubayashi, N. Kitazawa, A. Shomura, K. Matsubara, N. Ono, R. Mizobuchi, T. Shibaya et al. (2015) Advanced backcross QTL analysis reveals complicated genetic control of rice grain shape in a *japonica* × *indica* cross. Breed Sci 65: 308–318. 26366113 10.1270/jsbbs.65.308 PMC4542931

[B15] Nagata, K., Y. Nonoue, K. Matsubara, R. Mizobuchi, N. Ono, T. Shibaya, K. Ebana, E. Ogiso-Tanaka, T. Tanabata, K. Sugimoto et al. (2023) Development of 12 sets of chromosome segment substitution lines that enhance allele mining in Asian cultivated rice. Breed Sci 73: 332–342. 37840983 10.1270/jsbbs.23006 PMC10570878

[B16] Ngangkham, U., M. Nath, P. Dokku, S.V. Amitha Mithra, S. Ramamurthy, N.K. Singh, R.P. Sharma and T. Mohapatra (2018) An EMS-induced new sequence variant, TEMS5032, in the coding region of SRS3 gene leads to shorter grain length in rice (*Oryza sativa* L.). J Appl Genet 59: 377–389. 30014258 10.1007/s13353-018-0455-4

[B17] Ogawa, D., T. Sakamoto, H. Tsunematsu, N. Kanno, Y. Nonoue and J.I. Yonemaru (2021a) Haplotype analysis from unmanned aerial vehicle imagery of rice MAGIC population for the trait dissection of biomass and plant architecture. J Exp Bot 72: 2371–2382. 33367626 10.1093/jxb/eraa605 PMC8006554

[B18] Ogawa, D., T. Sakamoto, H. Tsunematsu, N. Kanno, Y. Nonoue and J.I. Yonemaru (2021b) Remote-sensing-combined haplotype analysis using multi-parental advanced generation inter-cross lines reveals phenology QTLs for canopy height in rice. Front Plant Sci 12: 715184. 34721450 10.3389/fpls.2021.715184 PMC8553969

[B19] Shi, H., P. Yun, Y. Zhu, L. Wang, Y. Wang, P. Li, H. Zhou, S. Cheng, R. Liu, G. Gao et al. (2024) Natural variation of *WBR7* confers rice high yield and quality by modulating sucrose supply in sink organs. Plant Biotechnol J 22: 2985–2999. 38943653 10.1111/pbi.14420 PMC11501006

[B29] Sumiyoshi, M., A. Nakamura, H. Nakamura, M. Hakata, H. Ichikawa, H. Hirochika, T. Ishii, S. Satoh and H. Iwai (2013) Increase in cellulose accumulation and improvement of saccharification by overexpression of arabinofuranosidase in rice. PLoS One 8: e78269. 24223786 10.1371/journal.pone.0078269 PMC3817243

[B20] Takai, T., T. Ikka, K. Kondo, Y. Nonoue, N. Ono, Y. Arai-Sanoh, S. Yoshinaga, H. Nakano, M. Yano, M. Kondo et al. (2014) Genetic mechanisms underlying yield potential in the rice high-yielding cultivar Takanari, based on reciprocal chromosome segment substitution lines. BMC Plant Biol 14: 295. 25404368 10.1186/s12870-014-0295-2 PMC4243286

[B21] Takehara, K., K. Murata, T. Yamaguchi, K. Yamaguchi, G. Chaya, S. Kido, Y. Iwasaki, H. Ogiwara, T. Ebitani and K. Miura (2018) Thermo-responsive allele of *sucrose synthase 3* (*Sus3*) provides high-temperature tolerance during the ripening stage in rice (*Oryza sativa* L.). Breed Sci 68: 336–342. 30100800 10.1270/jsbbs.18007 PMC6081304

[B22] Tanksley, S.D. and J.C. Nelson (1996) Advanced backcross QTL analysis: a method for the simultaneous discovery and transfer of valuable QTLs from unadapted germplasm into elite breeding lines. Theor Appl Genet 92: 191–203. 24166168 10.1007/BF00223376

[B23] Wang, E., J. Wang, X. Zhu, W. Hao, L. Wang, Q. Li, L. Zhang, W. He, B. Lu, H. Lin et al. (2008) Control of rice grain-filling and yield by a gene with a potential signature of domestication. Nat Genet 40: 1370–1374. 18820698 10.1038/ng.220

[B24] Yamakawa, H., T. Ebitani and T. Terao (2008) Comparison between locations of QTLs for grain chalkiness and genes responsive to high temperature during grain filling on the rice chromosome map. Breed Sci 58: 337–343.

[B25] Yamamoto, T., J. Yonemaru and M. Yano (2009) Towards the understanding of complex traits in rice: substantially or superficially? DNA Res 16: 141–154. 19359285 10.1093/dnares/dsp006 PMC2695773

[B26] Yoshioka, Y., H. Iwata, M. Tabata, S. Ninomiya and R. Ohsawa (2007) Chalkiness in rice: potential for evaluation with image analysis. Crop Sci 47: 2113–2120.

[B27] Zhao, D., C. Zhang, Q. Li and Q. Liu (2022) Genetic control of grain appearance quality in rice. Biotechnol Adv 60: 108014. 35777622 10.1016/j.biotechadv.2022.108014

