# Supplementary material for: Selection of chromosome segment substitution lines with reduced grain chalkiness without yield penalty in rice
Source: Breed Sci. 2025 Mar 22;75(2):79–84. doi: 10.1270/jsbbs.24044 (PMC12395195; doi:10.1270/jsbbs.24044)
Supplement: Supplementary file 1 — Supplemental Figures [file 75_079_s1.pdf]

Fukuda et al./Breeding Science
doi: 10.1270/jsbbs.24044

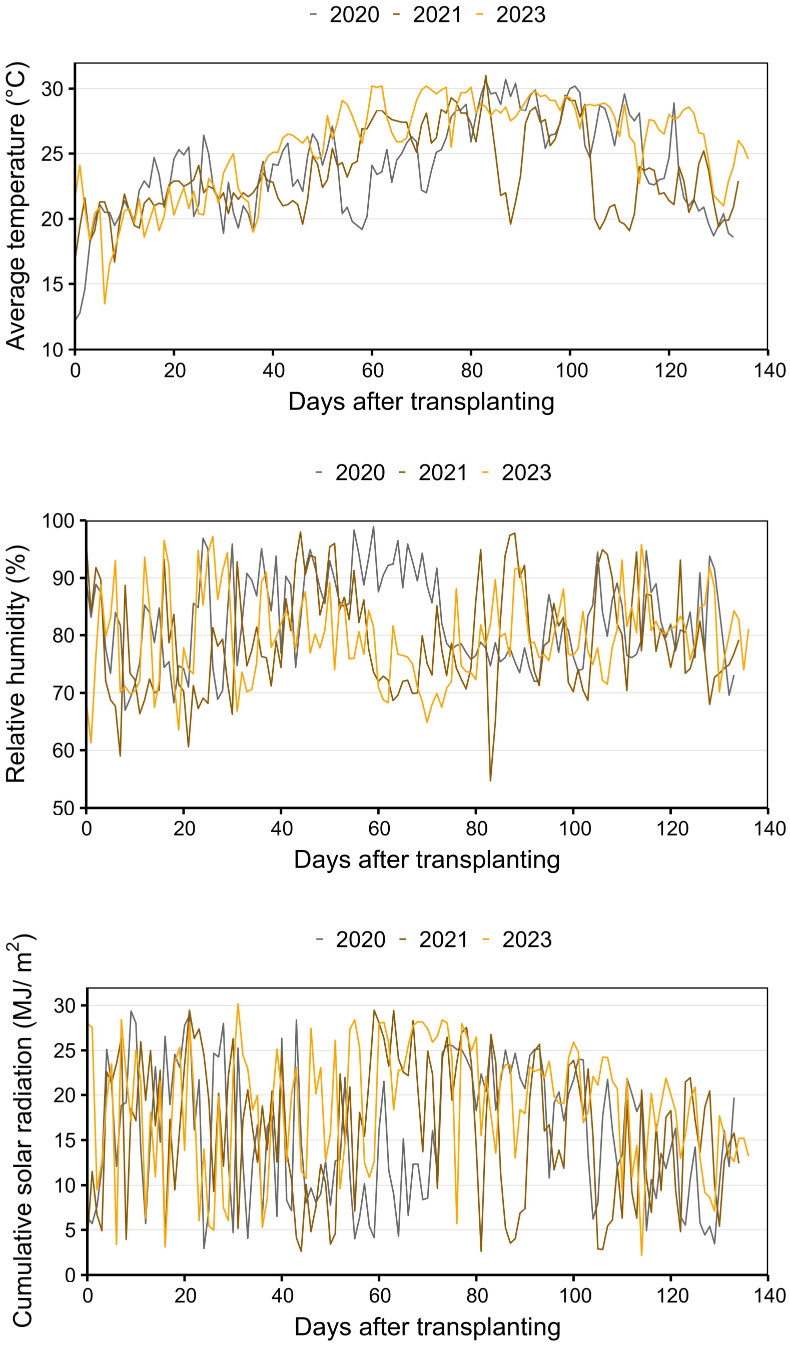

Supplemental Fig. 1. Mean daily temperature, humidity, and solar radiation from the date of transplanting (May) to the end of September in 2020, 2021 and 2023.

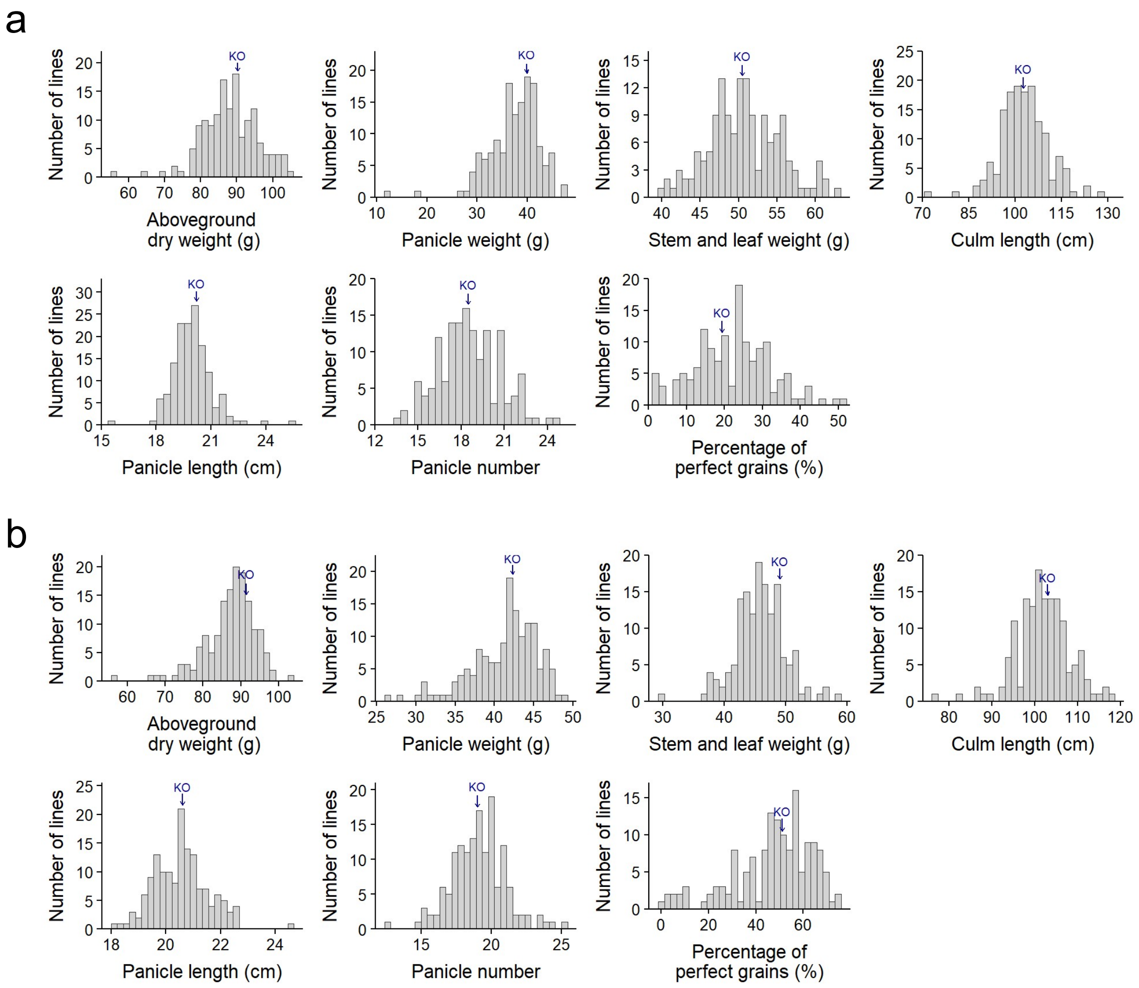

Supplemental Fig. 2. Phenotypic diversity of CSSLs and 'Koshihikari' in 2020 and 2021.

Histograms of phenotypic values in all 148 CSSLs and 'Koshihikari' (KO, blue arrow) grown in a paddy field in (a) 2020 and (b) 2021.

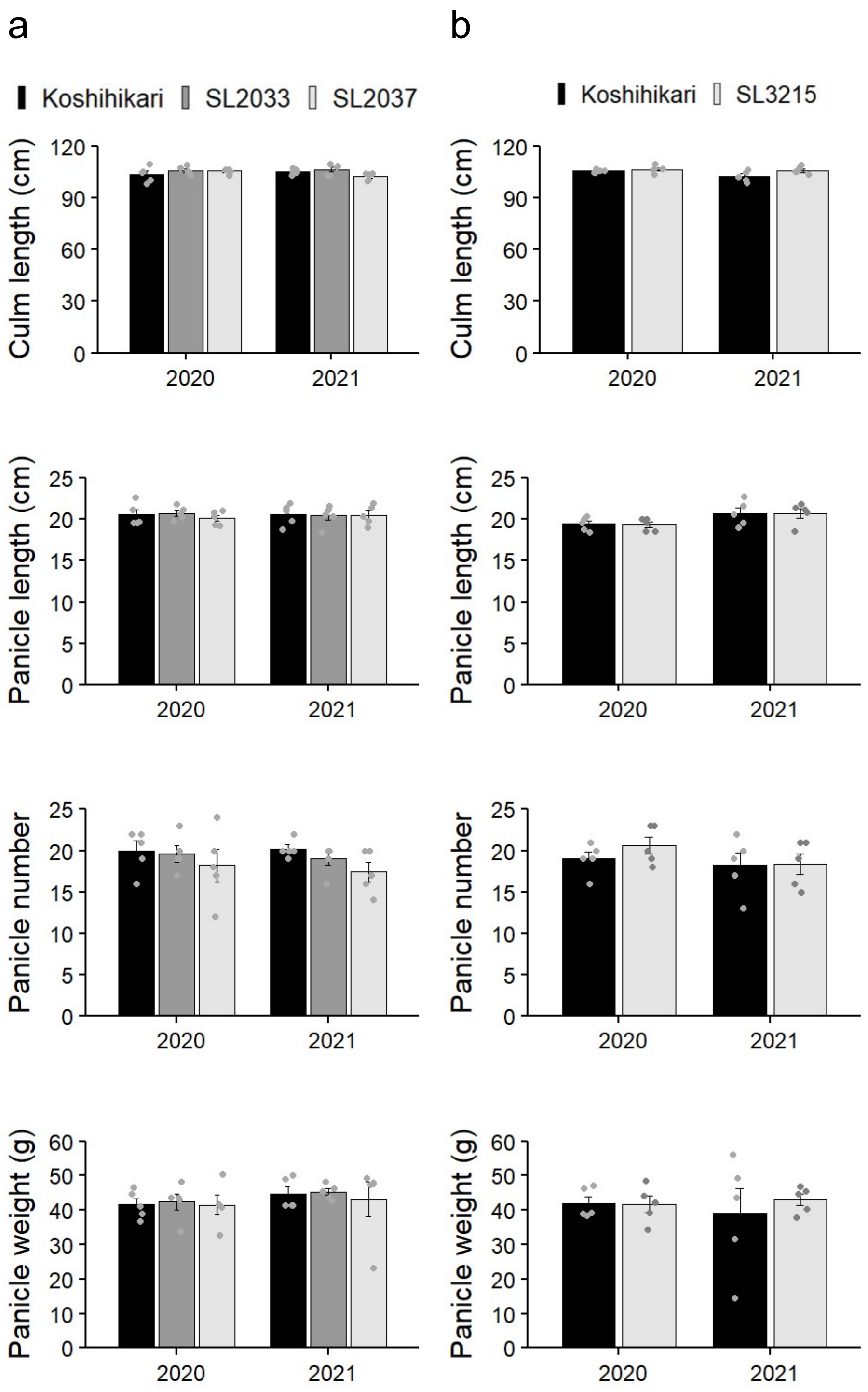

Supplemental Fig. 3. Comparison of agronomic traits between CSSLs and 'Koshihikari' cultivated in 2020 and 2021.
Culm length, panicle length, panicle number, and panicle weight in CSSLs (SL2033, SL2037, and SL3215) and 'Koshihikari'. 'Koshihikari' plants for each comparison were selected from the plants grown nearest each CSSL in 2020 and 2021. Values are individual data points and means ± SE (n = 5). There were no significant differences between each CSSL and 'Koshihikari' ($P > 0.05$, Dunnett's test or Student t-test).
